# Brilliant Reception

**Published:** 1881-07-20

**Authors:** 


### EDITORIALS AND MISCELLANEOUS.

### *BRILIANT RECEPTION.*

The reception given by Drs. McCaw and McGuire, at the Westmoreland Club-house, to the members of the American Medical Association (says a Richmond paper), was an occasion which will long be remembered with pleasure by those in attendance—the number being in all about six hundred and fifty. From 9 o'clock until 12, the parlors of the admirably arranged building was thronged with an assemblage as brilliant as ever gathered together in Richmond on a similar occasion. Music and flowers added to the attractiveness of the scene. The full orchestra, under the *baton* of Prof. Reinhardt, rendering choice selections during the evening.

Among the distinguished physicians present were Dr. Brodie, of Detroit; Dr. Agnew, of Philadelphia; Dr. Percival, of Maryland, and Dr. Sayre, of New York.

The entertainment given by Dr. and Mrs. R. T. Coleman, to the visiting physicians, was a most brilliant affair, and will ever be kindly and pleasantly remembered. We regret the want of space to give full expression in regard to it, and to the many hospitalities so characteristic of Old Virginia, which were extended to the members of the Association.

